# 17β-Hydroxysteroid dehydrogenases 1 and 2: potential markers for breast cancer recurrence and tamoxifen resistance among premenopausal women diagnosed with breast cancer in Denmark

**DOI:** 10.1186/s13058-025-02196-2

**Published:** 2025-12-30

**Authors:** Lindsay J. Collin, Kirsten M. Woolpert, Anders Kjaersgaard, Thomas P. Ahern, Michael Goodman, Lauren E. McCullough, Lance A. Waller, Kristina B. Christensen, Per Damkier, Stephen J. Hamilton-Dutoit, Kristina L. Lauridsen, Peer M. Christiansen, Bent Ejlertsen, Henrik T. Sørensen, Deirdre P. Cronin-Fenton, Timothy L. Lash

**Affiliations:** 1 Department of Epidemiology, Rollins School of Public Health, Emory University, Atlanta, GA USA; 2 Department of Clinical Epidemiology, Center for Population Medicine, Department of Clinical Medicine, Aarhus University and Aarhus University Hospital, Aarhus, Denmark; 3 Department of Surgery, The Robert Larner, M.D. College of Medicine at The University of Vermont, Burlington, VT USA; 4 Department of Biostatistics and Bioinformatics, Rollins School of Public Health, Emory University, Atlanta, GA USA; 5 Department of Pathology, Aarhus University Hospital, Aarhus, Denmark; 6 Department of Clinical Pharmacology, Odense University Hospital, Odense, Denmark; 7 Department of Clinical Medicine, University of Southern Denmark, Odense, Denmark; 8 Breast Unit, Aarhus University Hospital/Randers Regional Hospital, Aarhus Denmark and on behalf of the Danish Breast Cancer Group, Aarhus, Denmark; 9 Danish Breast Cancer Group, Department of Oncology, Copenhagen University Hospital - Rigshospitalet, Copenhagen, Denmark; 10 Department of Clinical Medicine, University of Copenhagen, Copenhagen, Denmark

**Keywords:** Breast cancer, Recurrence, Biomarkers, Endocrine therapy

## Abstract

**Background:**

Premenopausal women diagnosed with estrogen receptor (ER)-positive breast cancer are prescribed 5–10 years of tamoxifen therapy to prevent or delay a recurrence. The enzymes 17β-hydroxysteroid dehydrogenase 1 and 2 (HSD17B1 and HSD17B2, respectively) regulate the relative concentrations of estrogen metabolites and may modify tamoxifen effectiveness. We evaluated the prognostic and predictive value of HSD17B1 and HSD17B2 expression in breast tumors.

**Methods:**

We identified a cohort of premenopausal breast cancer patients from the Danish Breast Cancer Group database (2002–2011) and stratified on ER status and receipt of tamoxifen (4600 ER+/TAM + and 1359 ER−/TAM−). Biomarkers HSD17B1 and HSD17B2 were assayed by immunohistochemistry. We used Cox proportional hazards regression to compute the hazard ratios (HRs) and 95% simulation intervals (SIs) associating each biomarker with recurrence, with bias adjustment to account for mismeasurement of biomarker expression and baseline selection bias from tumor sample availability.

**Results:**

Among ER+/TAM + breast cancers, 24% had any HSD17B1 expression compared with 13% of ER−/TAM − breast cancers. In the bias-adjusted analyses, women diagnosed with tumors positive for HSD17B1 expression had a slight increased rate of recurrence: HR = 1.13 (95% SI 0.90, 1.43) in the ER+/TAM + stratum and HR = 1.15 (95% SI 0.77, 1.79) in the ER−/TAM − stratum. A 10-unit increase in HSD17B2 expression corresponded with a decrease in the estimated rate of recurrence among ER+/TAM + patients (HR = 0.85, 95% SI 0.69, 1.05), but not among ER−/TAM − patients (HR = 1.07, 95% SI 0.82, 1.42).

**Conclusions:**

HSD17B1 may be a prognostic marker for recurrence and HSD17B2 may be predictive of response to tamoxifen therapy in breast cancer.

**Supplementary Information:**

The online version contains supplementary material available at 10.1186/s13058-025-02196-2.

## Introduction

Early detection and multimodal treatment protocols for breast cancer have contributed to a 5-year relative survival of approximately 90% in industrialized nations [1]. Despite this largely favorable relative survival, an estimated 20–40% of breast cancer patients will experience a recurrence, depending on their prognostic profile, and recurrence risk extends beyond 20 years after the initial diagnosis [2, 3]. 

More than two-thirds of premenopausal breast cancer patients are diagnosed with estrogen receptor-positive (ER+) disease [1, 4]. Estrogen and estrogen-related metabolites play a crucial role in the growth and proliferation of these hormone-dependent tumors [4]. To reduce the risk of recurrence, premenopausal women diagnosed with ER + breast cancer receive tamoxifen as an adjuvant endocrine therapy for 5–10 years after their initial diagnosis [5]. Tamoxifen is a selective estrogen receptor modulator, with metabolites that have strong affinity to the ER and therefore compete with endogenous estrogen metabolites, most notably estradiol, at the receptor-binding site [6]. The most active tamoxifen metabolite is endoxifen, which binds to the ER with 100× affinity compared with tamoxifen itself [6]. 

Estrogen synthesis in premenopausal women is primarily driven by estrogen production in the ovaries through the sulfatase pathway, which involves the conversion of dehydroepiandrosterone (DHEA) sulfate into DHEA and estrone sulfate into estrone by the sulfatase enzyme [4, 7]. The conversion of estrogen to its active form is largely regulated by two enzymes, 17β-hydroxysteroid dehydrogenases 1 and 2 (HSD17B1 and HSD17B2, respectively), which carry out both the catalytic conversion of estrone to estradiol and the reverse reaction [8]. This conversion occurs within the target cells, where the estrogenic effect is exerted via the ER. Tissue-specific concentrations of estrogen metabolites are determined by the relative expression of enzymes and availability of substrates [9]. The two enzymes, HSD17B1 and HSD17B2, may serve as therapeutic targets in women whose tumors are not responsive to tamoxifen [9–11]. Both enzymes have tissue-specific expression, making them valid candidates for targeted therapy.

Breast tumors with increased expression of HSD17B1 are thought to have a corresponding increase in intratumoral concentrations of estradiol, thereby potentially reducing the effectiveness of tamoxifen through estradiol concentration [9–11]. Conversely, breast tumors with increased expression of HSD17B2 may have decreased intratumoral concentrations of estradiol, potentially improving the effectiveness of tamoxifen.

No study has evaluated the association of HSD17B1 and HSD17B2 expression with breast cancer recurrence in premenopausal women. To address this knowledge gap, we evaluated the predictive (i.e., indicative of tamoxifen therapy effectiveness) and/or prognostic (i.e., indicative of recurrence risk) roles of HSD17B1 and HSD17B2 expression in breast cancer recurrence using data from a large population-based cohort of exclusively premenopausal Danish women.

## Methods

### Study population

The Predictors of Breast Cancer Recurrence (ProBe CaRe) cohort study consists of 5959 premenopausal women diagnosed with a first primary stage I–III breast cancer between January 1, 2002 and December 31, 2011 in Denmark [12]. Of the 8047 premenopausal women diagnosed with breast cancer during the study period and recorded in the Danish Breast Cancer Group (DBCG) clinical database, 5959 patients without neoadjuvant therapy were identified as eligible based on their cancer stage at diagnosis. Study participants were placed into two groups classified by ER expression (defined as >1% positive staining of tumor cells) and receipt of tamoxifen (TAM). The study population consisted of 4600 ER+/TAM + and 1359 ER−/TAM − breast cancer patients.

During our study period, women diagnosed with breast cancer and subsequently enrolled in the DBCG underwent semi-annual examinations during the first five years following diagnosis and annual examinations in years 6–10 [13]. Follow-up for cohort members in the current study extended from breast cancer surgery until the first of (a) breast cancer recurrence, (b) death, (c) ten years of follow-up, (d) loss to follow-up due to emigration, (e) diagnosis of another malignancy, or (f) end of the study follow-up period (December 31, 2017). Mortality and emigration were identified using the Danish Civil Registration System, which is updated daily [14]. In this study, breast cancer recurrence is defined according to DBCG guidelines, which include any contralateral or ipsilateral breast cancer occurring locally, regional metastasis, or distant metastasis, after the initial breast cancer diagnosis [15].

### Data collection from Danish health registries

At birth or upon immigration, each resident of Denmark is assigned a unique 10-digit Civil Personal Registration (CPR) number, which allows individual-level linkage across Danish registries [14]. Leveraging this system we were able to abstract data from the DBCG clinical database, including information on demographics (age, menopausal status, and hospital issuing the diagnosis), tumor characteristics (stage, histological grade, lymph node involvement, and ER and human epidermal growth factor receptor-2 [HER2] expression), and treatment characteristics (primary surgical tumor management, planned radiation therapy, chemotherapy, anti-HER2 therapy, and tamoxifen therapy).

### Tissue microarray construction and immunohistochemistry

In Denmark, paraffin blocks from pathology specimens are routinely archived after diagnosis. Patient CPR numbers were used to link the study population to the Danish Pathology Data Bank, enabling us to locate tumor blocks for approximately 86% of study patients. For these patients, formalin-fixed, paraffin-embedded (FFPE) primary tumor tissue blocks were obtained from the pathology archives of treating hospitals. In total, we received 5116 tumor blocks, of which 4684 (79%) were used for construction of tissue microarrays (TMA) and 432 were excluded due to non-identifiable tumor samples (*n* = 405), megablocks unsuitable for construction (*n* = 13), and requests for the return of the blocks before the TMA could be constructed (*n* = 14, likely in order to inform clinical care). Laboratory personnel were blinded to all clinical information including ER status, recurrence status, and receipt of tamoxifen therapy.

TMAs were constructed using standard techniques. A section was cut from each study participant’s paraffin block and stained with hematoxylin and eosin. The diagnosis was confirmed by a study pathologist. Areas containing invasive breast carcinoma were identified and marked. Then core samples (1 mm diameter) were removed from each tumor donor block and re-embedded in a new recipient paraffin TMA block using the TMA Grand Master (3DHISTECH, Budapest, Hungary). If sufficient material was available, three representative tumor cores were sampled. Colon and kidney cores were included in each TMA to facilitate orientation within the TMA during microscopy.

Immunohistochemistry (IHC) staining was performed on the 3 μm thick TMA section cores. Both HSD17B1 and HSD17B2 were stained on a Ventana Benchmark Ultra automated stainer (Roche Diagnostics). To assay HSD17B1 expression, we used a rabbit monoclonal antibody at a concentration of 1:250 (Abcam ab51045), diluted with Dako REAL antibody diluent. To assay HSD17B2 expression, we used a rabbit polyclonal antibody at a concentration of 1:200 (PTGlab 10978-1-AP), diluted with Dako REAL antibody diluent. Both were incubated for 32 min at room temperature, and heat-induced epitope retrieval was performed using CC1 for 32 min. The slides were counterstained using hematoxylin II for 8 min. All slides were scanned using a Hamamatsu Nanozoomer 2.0 HT (Hamamatsu Photonics, Hamamatsu, Japan) scanner and visualization of expression was achieved using the Optiview DAB detection system (Ventana, Roche Diagnostics).

### TMA core scoring

**Fig. 1:**
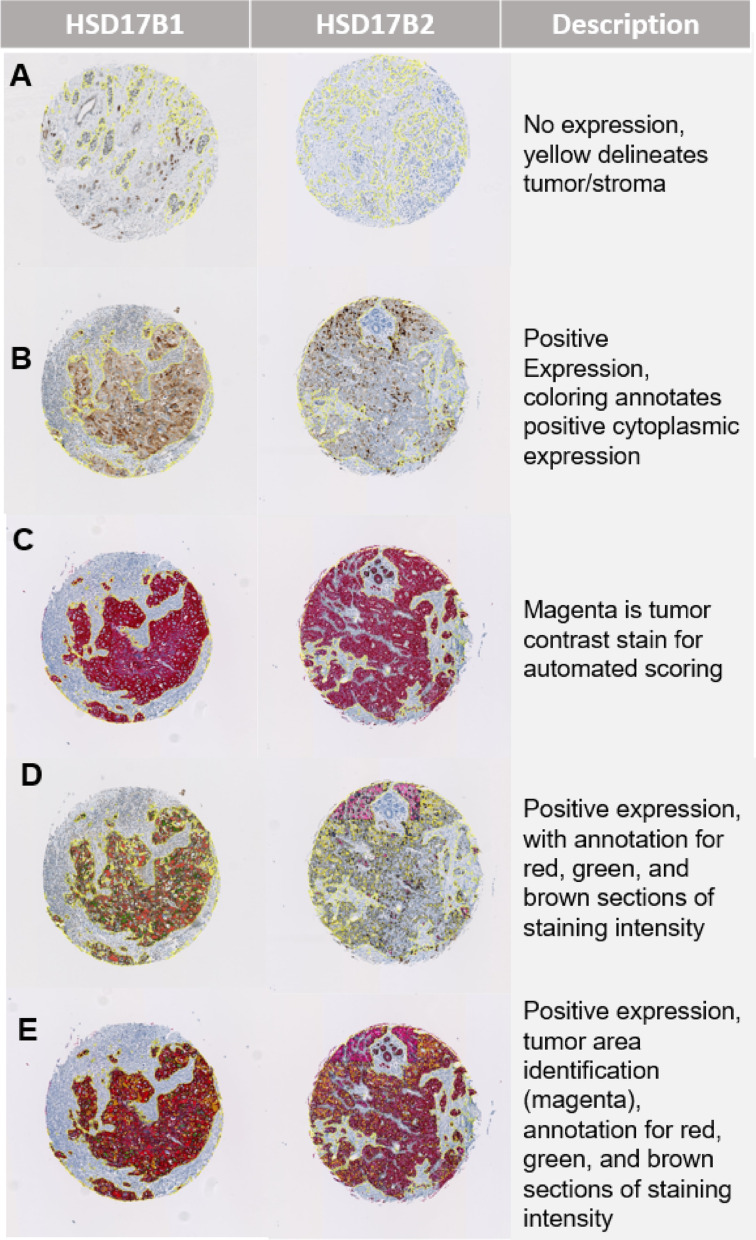
Illustration of the automated scoring algorithm for HSD17B1 and HSD17B2 for (**A**) no expression, (**B**) positive expression, (**C**) positive expression with tumor/stroma delineation, (**D**) positive expression with annotation of areas with low, medium and high intensity tumor stain, and (E) tumor stain with intensity annotation. Cores are magnified to 40x and have not been adjusted in any other way

Expression of HSD17B1 and HSD17B2 was quantified using automated scoring technology from Visiopharm (Hoersholm, Denmark). Expression of each biomarker was based on the relative area of tumor cores that met criteria for low, medium, or high staining intensity (Fig. 1). The combined scores generated from the automated scoring application were then merged into a single H-score, which itself was a combination of staining intensity and relative positive tumor area [16]. Staining intensity was characterized using a weighted scale ranging from 0 for no staining to 3 for high staining. The H-score therefore has a plausible range from 0 to 300. Each specimen had 1–3 available tumor cores. Most specimens had 3 tumor cores available (84% for ER+/TAM + and 83% for ER−/TAM−). We used the average H-score of the assayable cores as a measure of biomarker expression for each patient tumor. In a sensitivity analysis, we used percent positivity, which was derived as an area percentage ranging from 0 to 100%, based on the relative area of positively stained tumor cells.

### Exposure assessment

The biomarkers were assessed within ER/TAM strata, which allowed for evaluation of the enzymes as predictive and prognostic markers. If the biomarkers are predictive of tamoxifen resistance, then we would expect to observe an association in the ER+/TAM + stratum, but not in the ER−/TAM − stratum. If we observed an association in both strata, this would suggest that the biomarkers affect breast cancer recurrence directly (i.e., as a prognostic marker).

Due to the low observed staining prevalence of HSD17B1, we dichotomized HSD17B1 expression to any vs. none, defined as an H-score ≥ 1 vs. <1 (percent positivity ≥ 1% vs. <1% in the sensitivity analysis). HSD17B2 was expressed in most tumor samples. Thus, we evaluated HSD17B2 as a continuous exposure and as a categorical variable using quartiles of the H-scores of HSD17B2 (and percent positivity in the sensitivity analysis). We observed a threshold effect, in which the estimates for quartiles 2, 3, and 4 had nearly the same estimate of association. Thus, we combined quartiles 2, 3, and 4 to create a dichotomous variable, which was compared with the lowest quartile of HSD17B2.

### Validation study

To validate the automated scoring against gold-standard scores assigned by a pathologist, we sampled tumor cores for each biomarker using several approaches. First, to ensure that validation data were collected from different hospitals, we randomly sampled 5 hospitals among those that contributed at least 200 patients to the study. Second, we randomly selected one TMA from each of the 5 hospitals. Finally, we randomly sampled 100 patients from the 5 selected TMAs. The same patients were used to validate both biomarkers. This sampling selected 50 patients from each ER/TAM stratum. From the validation data for continuous HSD17B2 expression, we calculated the correlation coefficient between the automated scoring application and pathologist scores and applied this to regression calibration to account for measurement error [17, 18]. We additionally computed the sensitivity and specificity estimates using the dichotomous HSD17B2 exposure variable.

For HSD17B1, the validation data were sparse, and the estimates of sensitivity and specificity were incompatible with the cohort data (i.e., produced negative cell counts in the quantitative bias analysis). Therefore, we augmented the validation sample for HSD17B1 by sampling an additional 100 patients to increase the sample sizes within strata of observed HSD17B1 status (H-score ≥ 1 vs. <1) and recurrence across the strata. This strategy mimicked a balanced design [19]. Then, using the augmented validation data and the sampling weights, we computed estimates of sensitivity and specificity within strata of ER/TAM.

### Statistical analysis

We first described the distributions of the covariates of interest within each ER/TAM stratum. We reported the distribution of cytoplasmic HSD17B1 and HSD17B2 expression, providing information with respect to the baseline cohort and the exposure variables of interest for the observed data.

We presented unadjusted 10-year cumulative incidence of recurrence curves within ER/TAM strata of HSD17B1 expression (≥ 1 vs. < 1) and of the dichotomized HSD17B2 expression [20]. We fit Cox proportional hazards models to calculate the hazard ratios (HRs) and 95% confidence intervals (CIs) associating HSD17B1 and HSD17B2 expression with breast cancer recurrence. We presented both unadjusted models and models adjusting for age at diagnosis, cancer stage, surgery type, radiation therapy, and receipt of chemotherapy. Analyses were performed within ER/TAM strata to evaluate the predictive and/or prognostic utility of the biomarkers.

### Quantitative bias analysis

Our primary results were based on quantitative bias analysis, since our study may be susceptible to systematic errors. These errors include mismeasurement of biomarker expression and selection bias from approximately 20% of participants for whom tumor blocks were unavailable. To account for these systematic errors, we performed a multiple bias analysis in a two-step process. First, as the exposure variable was missing for 20% of patients without available tumor samples, we used multiple imputation to assign the missing exposure status [21]. To support missing data imputation, we evaluated the distribution of covariates by tumor availability and ER/TAM strata (Table S1). Breast cancer patients without available tumor samples were similar to those with tumor samples across strata of tumor and treatment characteristics, except that women without available tumor samples were more likely to be diagnosed with stage I breast cancer (30% vs. 25%). Differences in tumor availability are therefore likely missing at random. This pattern suggests that imputation can be used based on measured tumor, treatment, and patient characteristics [21]. Imputation was based on age, stage, tumor size, lymph node status, surgery type (mastectomy vs. breast conserving surgery), chemotherapy, radiation therapy, follow-up time, recurrence, grade, HER2 status, and treatment hospital. We performed 50 imputation sets and used mean values for both biomarkers. The number of imputation sets was based on the proportion missing in the overall sample so as to have sufficient number of imputation sets to produce reliable standard errors [22, 23].

Second, using the results from the internal validation substudy, we used probabilistic bias analysis and regression calibration to account for possible measurement error affecting the biomarkers. These bias adjustments were based on the classification of each biomarker using the automated scoring method compared with the gold-standard scoring performed by a pathologist. For HSD17B1, we assigned the sensitivity and specificity estimates and the bias-adjusted prevalence of exposure in the population to calculate positive and negative predictive values (PPV and NPV, respectively) [24, 25]. We assigned beta distributions to the PPV and NPV, informed by the validation data, which were then used in a record-level probabilistic bias analysis. We considered the continuous measure of HSD17B2 to be the exposure of interest and used regression calibration to account for measurement error of the continuous H-score. We additionally used the estimates of sensitivity and specificity in a probabilistic bias analysis with the dichotomous HSD17B2 measure. However, as the validation data were relatively sparse, the estimate for specificity was not compatible with the observed data in the ER−/TAM − stratum. For both HSD17B1 and HSD17B2, we performed 10,000 iterations in the bias analysis simulation, and within each iteration, we calculated the bias-adjusted HR and 95% simulation interval (SI). To incorporate conventional random error, we used bootstrap approximation [26]. The final reported estimates were the median and the 2.5th and 97.5th percentiles of the distribution of results generated for each biomarker. All analyses were carried out using R v4.4 (Vienna, Austria).

## Results

The ProBe CaRe cohort includes 4600 ER+/TAM + and 1359 ER−/TAM − premenopausal women diagnosed with breast cancer in Denmark. At diagnosis, ER+/TAM + breast cancer patients were older than ER−/TAM − patients (60% vs. 46% ≥45 years of age) and more likely to recommended radiation therapy as part of care (86% vs. 80%) than ER−/TAM − breast cancer patients (Table 1). Additionally, compared with women in the ER−/TAM − stratum, ER+/TAM + women were less likely to have node negative disease (37% vs. 51%) and to be diagnosed with HER2 + breast carcinomas (14% vs. 26%). Follow-up was longer among patients in the ER+/TAM + stratum, with median follow-up of 93 months (interquartile range (IQR): 71, 120 months) compared with 81 months (IQR: 47, 118 months) among patients in the ER−/TAM − stratum. With respect to the biomarkers of interest, the observed conventional prevalence estimates were 24% for ER+/TAM + breast cancer patients with HSD17B1 expression vs. 13% for ER−/TAM − patients. Women in the ER−/TAM − stratum were less likely to have expression in the lowest quartile of HSD17B2 expression (21% vs. 26%). Over the course of follow-up, we observed 566 recurrences (12%) among women in the ER+/TAM + stratum, and 265 recurrences (19%) among women in the ER−/TAM − stratum. Most recurrences were distant recurrences (70%: ER+/TAM + vs. 55% ER−/TAM−), with metastases to the bone being more common among ER+/TAM + patients (45%) than ER−/TAM − patients (22%) (Table S2).

**Table 1:** Distribution of clinical and tumor characteristics by ER status and receipt of Tamoxifen among the 5959 participants in a population-based cohort of premenopausal women diagnosed with a first primary breast cancer, probe care study, Denmark, 2002–2011

Patient and tumor characteristics	ER+/TAM+ (*N* = 4600)		ER–/TAM– (*N* = 1359)	
	Median	IQR	Median	IQR
Follow-up in months	93	71, 120	81	47, 118
Time to recurrence (months)	49	28, 75	26	14, 53

Patient and tumor characteristics	ER+/TAM+ (*N* = 4600)		ER–/TAM– (*N* = 1359)	
	N	%	N	%
*Cytoplasmic HSD17B1 Expression*				
Any Expression (≥ 1)	858	(24)	132	(13)
No Expression (< 1)	2699	(76)	853	(87)
Missing	1043		374	
*Cytoplasmic HSD17B2 Expression*				
≥ 25th %tile	2629	(74)	776	(79)
< 25th %tile	926	(26)	209	(21)
Missing	1045		374	
*Recurrence*				
Yes	657	(14)	283	(21)
No	3943	(86)	1076	(79)
*Site of Recurrence*				
Local	71	(11)	70	(25)
Regional	59	(9.0)	27	(9.5)
Distant	458	(70)	155	(55)
Contralateral	69	(11)	31	(11)
*Age at diagnosis*,* years*				
< 35	222	(4.8)	182	(23)
35–39	487	(11)	229	(27)
40–44	1123	(24)	321	(24)
45–49	1668	(36)	385	(28)
50+	1100	(24)	242	(18)
*UICC stage at diagnosis*				
Stage I	1184	(26)	402	(30)
Stage II	2476	(54)	702	(52)
Stage III	917	(20)	246	(18)
Unknown stage	23	(0.4)	9	(0.7)
*Tumor diameter*				
< 2 cm	2646	(58)	677	(50)
2 – <5 cm	1780	(39)	632	(47)
> 5 cm	156	(3)	44	(3)
Unknown	18	(0.4)	6	(0.4)
*Number of metastatic lymph nodes*				
0	1704	(37)	695	(51)
1	1148	(25)	238	(17)
2	583	(13)	116	(9)
3+	1152	(25)	306	(23)
*Histologic grade*				
Unsuitable	10	(0.2)	13	(1)
I	955	(21)	21	(1.5)
II	2391	(52)	216	(16)
III	950	(21)	884	(65)
Unknown	294	(6.4)	225	(17)
*Type of primary surgery*				
Mastectomy	2033	(44)	627	(46)
Lumpectomy	2567	(56)	732	(54)
*Progesterone receptor status*				
PR−	383	(8.3)	1121	(83)
PR+	2680	(58)	19	(1.4)
Unknown/Not measured	1537	(33)	219	(16)
*HER2 status*				
HER2+	2887	(63)	692	(51)
HER2−	619	(14)	354	(26)
Unknown/Not measured	1094	(24)	313	(23)
*Chemotherapy*				
Yes	4163	(91)	1250	(92)
No	437	(9.0)	109	(8)
*Radiation therapy*				
Yes	3945	(86)	1092	(80)
No	655	(14)	267	(20)
*Anti-HER2 therapy*				
Yes	619	(13)	354	(26)
No	2887	(63)	692	(51)
Unknown	1094	(24)	313	(23)

### Cumulative incidence curves

**Fig. 2:**
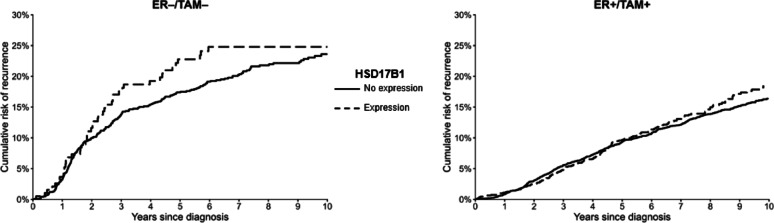
Unadjusted ten-year cumulative incidence plots for the imputed estimates of HSD17B1 expression in the ER+/TAM + and ER−/TAM −strata

**Fig. 3:**
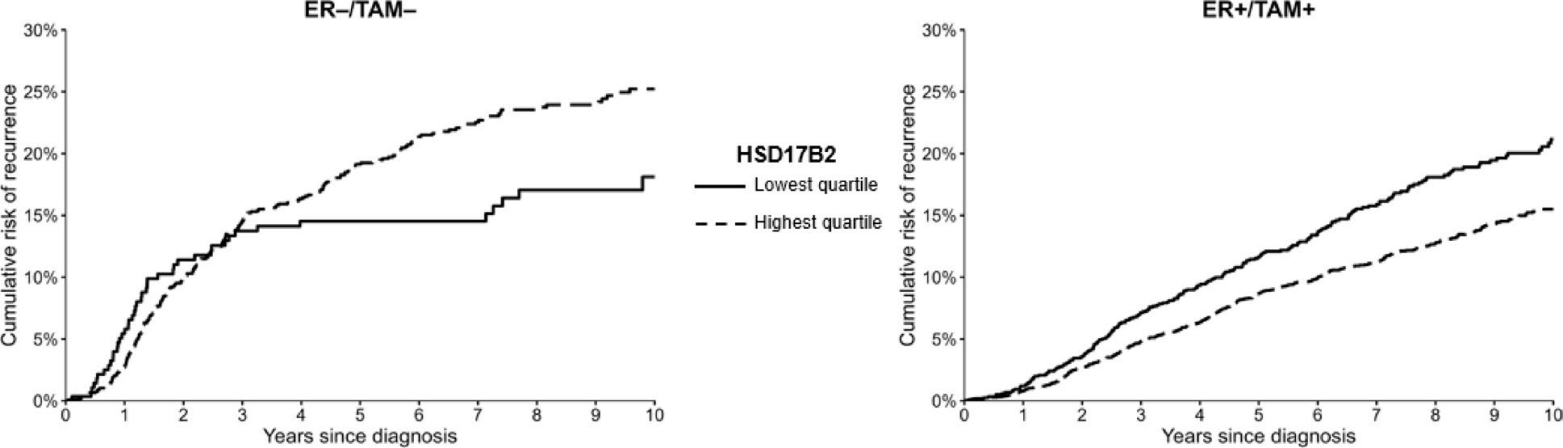
Unadjusted ten-year cumulative incidence plots for the imputed estimates of HSD17B2 expression comparing highest to lowest quartiles of expression in the ER+/TAM + and ER−/TAM−strata

The ten-year unadjusted cumulative risk estimates illustrate relatively similar recurrence risks for patients whose tumors had HSD17B1 expression vs. no expression by ER/TAM groups (Fig. 2). Among women in the ER+/TAM + stratum, those with any HSD17B1 expression had an increase in the cumulative risk of recurrence around 5 years after diagnosis, while the curves diverged around 2.5 years after follow-up among those in the ER−/TAM − stratum. The cumulative risk estimates comparing quartiles 2–4 with the lowest quartile of HSD17B2 expression suggested diverging curves at 2.5 years after diagnosis in the ER+/TAM + group (Fig. 3). In contrast, women in the ER−/TAM − stratum had an increase in the cumulative risk of recurrence in quartiles 2–4 of HSD17B2 expression.

### Validation substudy and quantitative bias analysis

Results from the validation substudy indicated that, on average, automated scoring underestimated biomarker expression compared with a gold-standard score assigned by a pathologist. For HSD17B1, the validation substudy indicated a low sensitivity of automated scoring ([60%, 95% CI 43%, 76%] and [59%, 95% CI 40%, 75%]) for ER+/TAM + and ER−/TAM−, respectively), and a high specificity ([96%, 95% CI 87%, 99%] and [98%, 95% CI 90%, 99%] for ER+/TAM + and ER−/TAM−, respectively). HSD17B2 expression also was underestimated with a correlation coefficient of 0.80.

In the analysis accounting for missing expression data and misclassification of HSD17B1, we observed a slight increase in the rate of recurrence (HR = 1.13; 95% SI 0.90, 1.43) in the ER+/TAM + stratum, comparing breast cancer patients with HSD17B1 tumor expression with those without (Table 2). This estimate was comparable to the multivariable-adjusted estimate of association observed in the conventional model, which did not account for missing expression data or potential misclassification of HSD17B1 (HR = 1.17, 95% CI 0.96, 1.43). Among women in the ER−/TAM − stratum, those with tumors positive for HSD17B1 also had a slightly higher breast cancer recurrence rate than women with tumors lacking HSD17B1 expression (HR 1.15, 95% SI 0.77, 1.79). This estimate also was similar to the conventional estimate (HR = 1.05, 95% CI 0.71, 1.54).

**Table 2:** Multivariable and bias-adjusted associations between cytoplasmic HSD17B1 and HSD17B2 expression and breast cancer recurrence among 4,599 subjects in the probe care premenopausal cohort study

	Events	Unadjusted HR (95%CI)	Multivariable-adjusted HR (95% CI)	Selection bias-adjusted HR (95% SI)	Multiple bias-adjusted HR (95% SI)
ER+/TAM+					
Cytoplasmic HSD17B1 expression					
Any Expression (H-score ≥ 1)	130	1.14 (0.94, 1.40)	1.17 (0.96, 1.43)	1.11 (0.93, 1.33)	1.13 (0.90, 1.43)
No Expression (H-score < 1)	371	Ref.	Ref.	Ref.	Ref.
Cytoplasmic HSD17B2 expression					
Continuous HSD17B2 (10-unit increase)	501	0.94 (0.90, 0.98)	0.96 (0.93, 1.00)	--	0.85 (0.69, 1.05)
Dichotomous HSD17B2 Expression					
≥ 25th %tile	276	0.73 (0.71, 0.74)	0.79 (0.77, 0.81)	0.77 (0.65, 0.91)	0.53 (0.19, 0.75)
< 25th %tile	166	Ref.	Ref.	Ref.	Ref.

ER−/TAM−					
Cytoplasmic HSD17B1 Expression					
Any Expression (H-score ≥ 1)	31	1.18 (0.80, 1.74)	1.05 (0.71, 1.54)	1.01 (0.73, 1.40)	1.15 (0.77, 1.79)
No Expression (H-score < 1)	167	Ref.	Ref.	Ref.	Ref.
Cytoplasmic HSD17B2 Expression					
Continuous HSD17B2 (10-unit increase)	198	1.02 (0.98, 1.06)	1.01 (0.97, 1.05)	--	1.07 (0.82, 1.42)
Dichotomous HSD17B2 Expression					
≥ 25th %tile	165	1.32 (1.26, 1.38)	1.26 (1.21, 1.32)	1.32 (0.95, 1.83)	NE
< 25th %tile	35	Ref.	Ref.	Ref.	

*HR* hazard ratio, *CI* confidence interval; *SI* simulation interva, *ER* estrogen receptor, *TAM* tamoxifen

For the association between HSD17B2 and breast cancer recurrence, accounting for missing expression data and measurement error, we observed that for a 10-unit increase in the HSD17B2 H-score the estimated rate of breast cancer recurrence was lower among women in the ER+/TAM + stratum (HR = 0.85, 95% SI 0.69, 1.05). This association was more pronounced than the conventional estimate (HR = 0.96, 95% CI 0.93, 1.00), suggesting a bias towards the null from the misclassification. In the ER−/TAM − stratum, the association for each 10-unit increase in HSD17B2 H-score was 1.07 (95% SI 0.82, 1.42)—similar to the conventional estimate (HR = 1.01, 95% CI 0.97, 1.05). In the analysis with dichotomous HSD17B2 expression, the bias-adjusted estimate was 0.53 (95% SI 0.19, 0.75) in the ER+/TAM + stratum, which was further from the null than the conventional estimate (HR = 0.79, 95% CI 0.77, 0.81). In the ER−/TAM − stratum, the conventional association indicated that HSD17B2 was associated with a higher estimated recurrence rate (HR = 1.26, 95% CI 1.21, 1.32). We were unable to compute the bias-adjusted estimate.

In the sensitivity analysis using percent positivity to quantify biomarker expression, the results were similar to those for the conventional analyses (Table S3).

## Discussion

In this study, we observed a modest association between HSD17B1 expression and breast cancer recurrence in both ER/TAM strata, indicating that the HSD17B1 biomarker may serve as a prognostic factor among premenopausal breast cancer patients. We also found that HSD17B2 expression may predict tamoxifen response among women diagnosed with ER + disease, with observed associations showing lower recurrence rates in the ER+/TAM + stratum and higher recurrence rates in the ER−/TAM − stratum. Our bias analysis generally revealed that selection bias due to missing tumor cores produced a bias away from the null, and that non-differential misclassification of exposure produced an expected bias towards the null. The results of the bias analysis were generally comparable to the conventional analysis, strengthening the validity of the observed associations, but the precision in the estimates decreased as we accounted for the additional layers of uncertainty.

The potential role of HSD17B1 in the development and progression of breast cancer has been investigated previously [27–29]. In vitro studies have suggested that HSD17B1 expression stimulates breast cancer cell growth through activation of estradiol and inactivation of dihydrotestostrone [10, 27–29]. In a population-based study, Gunnarsson et al. reported improved survival among postmenopausal ER + breast cancer patients with a HSD17B2:HSD17B1 ratio >0.2 compared with women with a HSD17B2:HSD17B1 ratio ≤ 0.2 [30]. The authors concluded that tamoxifen had a better clinical effect in patients with low HSD17B1 expression relative to high HSD17B2 expression. Consistent with our study results, they reported a worse prognosis among women diagnosed with ER + tumors and higher expression of HSD17B1 compared to women with lower HSD17B1 expression, and better prognosis among women diagnosed with ER + tumors with higher HSD17B2 expression compared to those with lower HSD17B2 expression. The Gunnarsson et al. study was conducted among postmenopausal women, whereas our study included only premenopausal women, for whom tamoxifen is recommended in current clinical guidelines, and in whom circulating estrogen concentrations are much higher [31]. 

The predictive or prognostic value of HSD17B2 expression has not been studied to the same extent as the value of HSD17B1 expression. HSD17B2 plays a role in the regulation of the relative concentrations of estradiol to estrone [32]. It is therefore biologically plausible that greater HSD17B2 expression in tumors occurring in premenopausal women would enhance tamoxifen treatment efficacy [19, 20]. The relative concentrations of estrone to estradiol are regulated by both HSD17B1 and HSD17B2 [33]. In our study, HSD17B2 expression appeared to be predictive of tamoxifen responsiveness among ER + tumors, which supports its importance in estradiol-stimulated cancer cell proliferation. However, we observed that higher expression of HSD17B2 was associated with higher recurrence in ER−/TAM − breast cancer patients. Previous studies have noted that HSD17B2 expression is higher in ER − tumors [34], but its role in cancer prognosis for ER − patients is not well understood. A previous in vitro analysis by Zhang et al. reported that mice that overexpressed HSD17B2 were more likely to develop ER−/TAM − breast cancers and concluded that HSD17B2 is both hormone- independent and hormone-dependent, which may explain our observed results [35]. 

This study has some important limitations. First, as detectable HSD17B1 expression had relatively low prevalence, estimates were imprecise in both the ER+/TAM + and ER−/TAM − groups due to the limited number of events among those whose tumors expressed the biomarker. Previous studies have reported 20% to 50% prevalence of HSD17B1 expression in ER + tumors. In our study, 24% of ER + tumors and 13% of ER − tumors expressed HSD17B1 by automated scoring. This increased to 42% and 25% when accounting for potential misclassification using the results from our internal validation substudy. Second, we were unable to incorporate information on changes in endocrine therapy from tamoxifen to aromatase inhibitors during the menopausal transition. Similarly, we did not account for adherence to endocrine therapy over the expected 5-year treatment duration, which may be important in future studies. Third, HSD17B1 and HSD17B2 regulate intratumoral concentrations of estrogen and related metabolites; however, we lacked information on the phenotypic expressions of these two enzymes and the corresponding concentrations of estradiol and estrone. Knowledge of the cellular concentrations of estradiol and estrone would provide additional insight into the underlying mechanism of the role of HSD17B1 and HSD17B2 in breast cancer recurrence and tamoxifen treatment efficacy. However, assay of intratumor concentrations of estradiol and estrone would be clinically impractical. The clinical utility of both biomarkers relates to their ability to be assessed using immunohistochemistry, which we have demonstrated here; however, RNA sequencing to measure mRNA expression levels directly may be informative in future studies to understand gene expression. Similarly, the aromatase enzyme converts androgens to estrogens, and has been linked to breast cancer treatment response and outcomes among premenopausal breast cancer patients, which we were unable to investigate in this study. Fourth, there may be misclassification of ER responsiveness given the cut-off of 1% to define ER-positive tumors. Finally, our study was conducted among Danish premenopausal breast cancer patients, a largely homogenous group. This may limit the generalizability of our study’s results if the biologic mechanism is not conserved across different racial and ethnic groups, although such heterogeneity seems unlikely. It is possible that the prevalence of biomarkers may differ across populations, but the relevance of the biomarkers would most likely be retained. Moreover, we lacked information on body mass index or obesity, which may influence estrogen concentration in our study population.

## Conclusions

Results from this study suggest that HSD17B1 expression may be a prognostic marker of breast cancer recurrence among premenopausal women. As well, HSD17B2 expression may confer responsiveness to tamoxifen among tamoxifen-treated premenopausal women diagnosed with ER + breast cancer. Future studies to evaluate the clinical utility of these biomarkers may be warranted.

## Supplementary Information

Below is the link to the electronic supplementary material.

Supplementary Material 1.

## Data Availability

The compilation and analysis of data in this study were conducted within the secure servers of Statistics Denmark and are not publicly available in accordance with Danish privacy laws. Procedures for accessing the data and a detailed study protocol can be made available by contacting the corresponding author.

## Abbreviations

ER: Estrogen receptor
DHEA: Dehydroepiandrosterone
HSD17B1: 17β-hydroxysteroid dehydrogenases 1
HSD17B2: 17β-hydroxysteroid dehydrogenases 2
DBCG: Danish Breast Cancer Group
TAM: Tamoxifen
CPR: Civil Personal Registration
HER2: Human epidermal growth factor receptor-2
FFPE: Formalin-fixed, paraffin-embedded
TMA: Tissue microarrays
HR: Hazard ratio
CI: Confidence interval
PPV: Positive predictive value
NPV: Negative predictive value
SI: Simulation interval
