# Supplementary material for: 17β-Hydroxysteroid dehydrogenases 1 and 2: potential markers for breast cancer recurrence and tamoxifen resistance among premenopausal women diagnosed with breast cancer in Denmark
Source: Breast Cancer Res. 2025 Dec 30;28:28. doi: 10.1186/s13058-025-02196-2 (PMC12857100; doi:10.1186/s13058-025-02196-2)
Supplement: Supplementary file 1 — Supplementary Material 1. [file 13058_2025_2196_MOESM1_ESM.docx]

| **TABLE S1.** Distribution of clinical and tumor characteristics by tumor sample availability within ER and receipt of tamoxifen (TAM) strata, among 5,959 participants in a population-based cohort of premenopausal women diagnosed with first primary breast cancer in Denmark (2002–2011), ProBe CaRe study. | | | | | | | | |
| --- | --- | --- | --- | --- | --- | --- | --- | --- |
|  | **ER+/TAM+** | | | | **ER−/TAM–** | | | |
| **Patient and tumor characteristics** | **Tumor Available** | | **No Tumor Available** | | **Tumor Available** | | **No Tumor Available** | |
|  | **N** | **%** | **N** | **%** | **N** | **%** | **N** | **%** |
| **Total** | 3596 | (78) | 1004 | (22) | 1003 | 74 | 356 | (26) |
| **Age at diagnosis** |  |  |  |  |  |  |  |  |
| <35 | 175 | (79) | 47 | (21) | 137 | (75) | 45 | (25) |
| 35–39 | 372 | (76) | 115 | (24) | 157 | (69) | 72 | (31) |
| 40–44 | 889 | (79) | 234 | (21) | 231 | (72) | 90 | (28) |
| 45–49 | 1323 | (79) | 345 | (21) | 284 | (74) | 101 | (26) |
| 50+ | 837 | (76) | 263 | (24) | 194 | (80) | 48 | (20) |
| **Stage at diagnosis** |  |  |  |  |  |  |  |  |
| Stage I | 884 | (75) | 300 | (25) | 276 | (69) | 126 | (31) |
| Stage II | 1957 | (79) | 519 | (21) | 540 | (77) | 162 | (23) |
| Stage III | 742 | (81) | 175 | (19) | 182 | (74) | 64 | (26) |
| Unknown stage | 14 | (57) | 10 | (43) |  |  |  |  |
| **Tumor diameter** |  |  |  |  |  |  |  |  |
| < 2mm | 1799 | (75) | 589 | (25) | 397 | (68) | 185 | (32) |
| 2 – <5mm | 1661 | (82) | 373 | (18) | 569 | (78) | 156 | (22) |
| > 5mm | 135 | (77) | 41 | (23) | 37 | (74) | 13 | (26) |
| **Number of metastatic lymph nodes** | |  |  |  |  |  |  |  |
| 0 | 1302 | (76) | 402 | (24) | 507 | (73) | 188 | (27) |
| 1 | 893 | (78) | 255 | (22) | 169 | (71) | 69 | (29) |
| 2 | 455 | (78) | 128 | (22) | 96 | (83) | 20 | (17) |
| 3+ | 938 | (81) | 214 | (19) | 229 | (75) | 77 | (25) |
| **Histologic grade** |  |  |  |  |  |  |  |  |
| Unsuitable | 4 | (40) | 6 | (60) | 2 | (15) | 11 | (85) |
| I | 758 | (79) | 197 | (21) | 19 | (90) | 2 | (9.5) |
| II | 1842 | (77) | 549 | (23) | 151 | (70) | 65 | (30) |
| III | 760 | (80) | 190 | (20) | 667 | (75) | 217 | (25) |
| Unknown | 232 | (68) | 62 | (32) | 164 | (73) | 61 | (17) |
| **Type of primary surgery** |  |  |  |  |  |  |  |  |
| Mastectomy | 1585 | (78) | 448 | (22) | 458 | (73) | 169 | (27) |
| Lumpectomy | 2011 | (78) | 556 | (22) | 545 | (74) | 187 | (26) |
| **Chemotherapy** |  |  |  |  |  |  |  |  |
| No | 340 | (78) | 97 | (22) | 72 | (66) | 37 | (34) |
| Yes | 3256 | (78) | 907 | (22) | 931 | (74) | 319 | (26) |
| **Radiation therapy** |  |  |  |  |  |  |  |  |
| No | 485 | (74) | 170 | (26) | 193 | (73) | 74 | (28) |
| Yes | 3111 | (79) | 834 | (21) | 810 | (74) | 282 | (26) |
| **Recurrence** |  |  |  |  |  |  |  |  |
| No | 3087 | (78) | 856 | (22) | 800 | (74) | 276 | (26) |
| Yes | 509 | (77) | 148 | (23) | 203 | (72) | 80 | (28) |

| **TABLE S2.** Distribution of continuous clinical and tumor characteristics by ER and receipt of tamoxifen among the 5,959 participants in a population-based cohort of premenopausal women diagnosed with first primary breast cancer in Denmark, ProBe CaRe study. | | | | |
| --- | --- | --- | --- | --- |
| **Patient and tumor characteristics** | **ER+/TAM+** | | **ER−/TAM−** | |
|  | **Median** | **IQR** | **Median** | **IQR** |
| **Follow-up in months** | 93 | 71, 120 | 81 | 47, 118 |
| **Time to event (months)** | 49 | 28, 75 | 26 | 14, 53 |
| **HSD17B1 H-score** | 0.08 | 0.02, 0.90 | 0.04 | 0.01, 0.20 |
| **HSD17B2 H-score** | 33 | 20.6, 49.0 | 42.2 | 23.5, 65.5 |
| **Site of Recurrence** | **N** | **%** | **N** | **%** |
| Local | 71 | (11) | 70 | (25) |
| Regional | 59 | (9.0) | 27 | (9.5) |
| Distant | 458 | (70) | 155 | (55) |
| Contralateral | 69 | (11) | 31 | (11) |
|  |  |  |  |  |
| Bones | 294 | (45) | 61 | (22) |
| Lymph Nodes | 85 | (13) | 53 | (19) |
| Residual mammary/contralateral | 129 | (9.1) | 88 | (31) |
| Lung | 40 | (6.1) | 38 | (13) |
| Liver | 51 | (7.8) | 14 | (5.0) |
| Other sites | 58 | (8.8) | 29 | (10) |

| **TABLE S3:** Multivariable associations between cytoplasmic HSD17B1 and HSD17B2 expression, as percent positivity, and breast cancer recurrence among 4599 subjects in the ProBe CaRe premenopausal cohort study. | | | |
| --- | --- | --- | --- |
|  | **ER+/TAM+** | | |
|  | **Events** | **Unadjusted HR (95%CI)** | **Adjusted HR (95%CI)** |
| **Cytoplasmic HSD17B1 Expression** |  |  |  |
| Any Expression (≥1) | 130 | 1.14 (0.94, 1.40) | 1.16 (0.95, 1.42) |
| No Expression (<1) | 371 | Reference | Reference |
|  |  |  |  |
| **Cytoplasmic HSD17B2 Expression** |  |  |  |
| Continuous HSD17B2 (10-unit increase) | 501 | 0.94 (0.90, 0.98) | 0.96 (0.93, 1.00) |
|  |  |  |  |
| ≥75th %tile | 92 | 0.68 (0.53, 0.87) | 0.79 (0.61, 1.02) |
| 50th–75th %tile | 118 | 0.72 (0.57, 0.91) | 0.78 (0.62, 0.99) |
| 25th–50th %tile | 129 | 0.75 (0.60, 0.95) | 0.79 (0.63, 1.00) |
| <25th %tile | 166 | Reference | Reference |
|  |  |  |  |
|  | **ER−/TAM−** | | |
| **Cytoplasmic HSD17B1 Expression** |  |  |  |
| Any Expression (≥1) | 31 | 1.20 (0.82, 1.76) | 1.09 (0.74, 1.60) |
| No Expression (<1) | 167 | Reference | Reference |
|  |  |  |  |
| **Cytoplasmic HSD17B2 Expression** |  |  |  |
| Continuous HSD17B2 (10-unit increase) | 198 | 1.02 (.98, 1.06) | 1.01 (0.97, 1.05) |
|  |  |  |  |
| ≥75th %tile | 83 | 1.45 (0.97, 2.17) | 1.40 (0.93, 2.08) |
| 50th–75th %tile | 42 | 1.16 (0.73, 1.82) | 1.12 (0.71, 1.76) |
| 25th–50th %tile | 40 | 1.31 (0.83, 2.07) | 1.21 (0.77, 1.92) |
| <25th %tile | 35 | Reference | Reference |
